# Divergent associations between adiposity and hernia subtypes in European populations: A Mendelian randomization study

**DOI:** 10.1097/MD.0000000000051209

**Published:** 2026-10-16

**Authors:** Yao Lu, Huiping Yuan, Ke Zhang, Dechun Liu

**Affiliations:** a Department of Thoracic Surgery, The First Affiliated Hospital, and College of Clinical Medicine of Henan University of Science and Technology, Luoyang, China; b Department of Endocrinology and Metabolism, The First Affiliated Hospital, and College of Clinical Medicine of Henan University of Science and Technology, Luoyang, China; c Department of Gastrointestinal Surgery, The First Affiliated Hospital, and College of Clinical Medicine of Henan University of Science and Technology, Luoyang, China.

**Keywords:** body mass index, causal inference, FinnGen, hernia, Mendelian randomization, obesity

## Abstract

Obesity is a suspected risk factor for hernia, but observational studies are susceptible to confounding and reverse causation. We aimed to investigate the causal effects of general and central adiposity on 5 hernia subtypes using Mendelian randomization (MR). Two-sample MR analyses were conducted using genetic instruments for 7 adiposity traits from the UK Biobank and hernia outcome data (diaphragmatic, femoral, inguinal, umbilical, and ventral hernias) from the Finn Gen consortium. The inverse-variance weighted method was the primary analysis, complemented by MR Egger and weighted median methods. Sensitivity analyses assessed heterogeneity, horizontal pleiotropy, and directionality. Outliers were removed in a confirmatory MR analysis. All *P* values were adjusted using the Benjamini–Hochberg (BH) method. Genetically predicted adiposity was associated with a decreased risk of inguinal hernia (e.g., body mass index [BMI] odds ratio [OR]: 0.84, 95% confidence interval [CI]: 0.76–0.92, *P*_BH_ <.001) but an increased risk of umbilical hernia (BMI OR: 1.39, 95% CI: 1.18–1.63, *P*_BH_ <.001) and ventral hernia (BMI OR: 1.93, 95% CI: 1.65–2.26, *P*_BH_ <.001). These effects were consistent across adiposity measures. No significant causal effects were found for diaphragmatic or femoral hernias. Sensitivity analyses confirmed the robustness of findings, with no horizontal pleiotropy and valid causal direction. This MR study suggests that genetically predicted adiposity has divergent potential causal associations with hernia subtypes, protecting against inguinal hernia while increasing the risk of umbilical and ventral hernias. These findings, though limited to European populations and subject to the assumptions of MR, have implications for clinical risk stratification and weight management counseling.

## 1. Introduction

Hernias, characterized by the protrusion of an organ or tissue through a weakness in the surrounding wall, represent a common and clinically significant surgical problem.^[1,2]^ Inguinal, femoral, umbilical, and ventral hernias are the most prevalent subtypes, each with distinct anatomical features and etiologies.^[3]^ While surgical repair is the definitive treatment, understanding the modifiable risk factors for hernia development is crucial for prevention and patient management.

Obesity, a global epidemic with rising prevalence, has long been implicated as a risk factor for hernia occurrence and recurrence.^[4,5]^ The proposed mechanisms include chronically increased intra-abdominal pressure (IAP), which strains the abdominal wall, and impaired tissue healing and collagen metabolism.^[6,7]^ However, observational epidemiological studies investigating the link between obesity and hernia are prone to confounding and reverse causation, making it difficult to establish a causal relationship.^[8]^ Furthermore, the impact of obesity might differ by hernia subtype, given their distinct pathophysiologies. For instance, while increased IAP might predispose to ventral hernias, the protective effect of a fat pad in the inguinal canal has also been hypothesized.^[9]^

Mendelian randomization (MR) offers a powerful approach to strengthen causal inference in such scenarios.^[8]^ MR uses genetic variants, which are randomly assorted at conception, as instrumental variables (IVs) for a modifiable exposure. This design mimics a randomized controlled trial and is less susceptible to confounding and reverse causation.^[10]^

Despite the established link between adiposity and hernia risk, critical knowledge gaps remain: the causal relationship between adiposity and hernia development has not been reliably confirmed, and whether adiposity confers homogeneous or divergent effects across different hernia subtypes is still unknown. Observational studies are limited by confounding and reverse causation, while no prior MR study has comprehensively evaluated multiple adiposity traits against a full spectrum of hernia subtypes. We therefore hypothesized that adiposity would exert distinct causal effects on different hernia subtypes owing to their divergent anatomical and pathophysiological features. Using a two-sample MR design, we systematically investigated the causal effects of seven adiposity-related traits (body mass index [BMI], body fat percentage, trunk fat percentage, trunk fat mass, whole-body fat mass, trunk fat-free mass, and whole-body fat-free mass) on 5 hernia subtypes (diaphragmatic, femoral, inguinal, umbilical, and ventral), aiming to clarify the causal role of adiposity in hernia etiology and subtype-specific risk heterogeneity.

## 2. Methods

### 2.1. Study design

We conducted a two-sample MR study using publicly available summary-level data from genome-wide association studies (GWAS) of European ancestry. The study design is illustrated in Figure 1. Ethical approval was not required for this study, as it involved secondary analysis of publicly available, de-identified, summary-level data from original studies that had each obtained the necessary approvals and participant consents as described in their respective publications. All ethical approval procedures for the included GWAS datasets were approved by the corresponding consortia, with full details documented in the original studies. The present MR analysis was conducted under 3 core assumptions, and their validity was empirically verified in this study: Relevance: Genetic variants were robustly associated with adiposity exposures, confirmed by *F*-statistics > 10 for all selected instruments; Independence: Genetic instruments were not associated with potential confounders, justified by the random allocation of alleles at conception and strict linkage disequilibrium clumping (*R*^2^ < 0.001) to minimize population stratification bias; Exclusion restriction: Genetic variants influenced hernia outcomes exclusively through adiposity, which was evaluated using the MR-Egger intercept test, Cochran’s *Q* heterogeneity test, Radial inverse-variance weighted (IVW) outlier removal, and the Steiger directionality test.^[11]^

**Figure 1.**
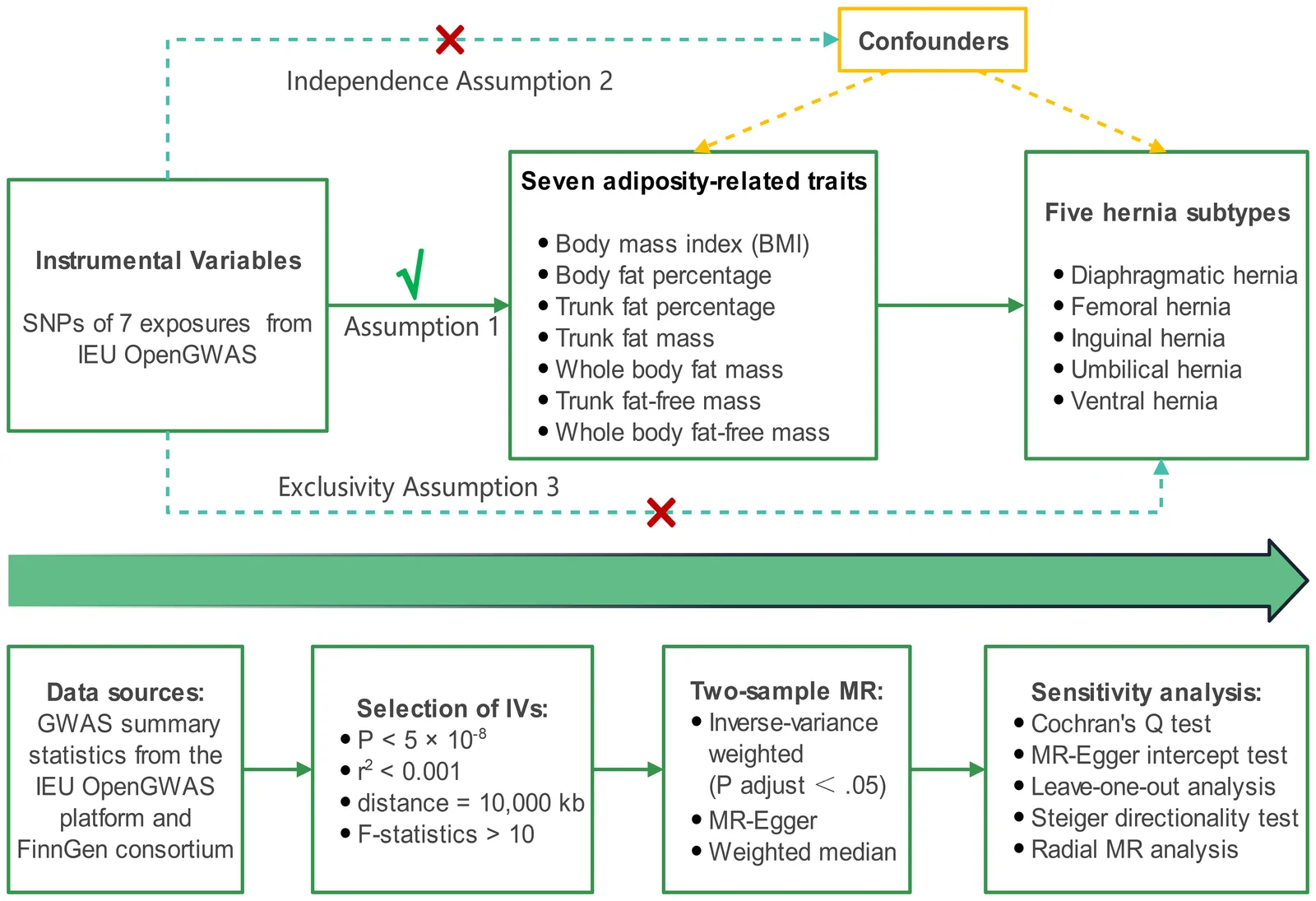
Study design overview. Schematic representation of the two-sample MR framework. Genetic variants (SNPs) associated with seven adiposity traits (exposures) were used as IVs to estimate causal effects on 5 hernia subtypes (outcomes). The analysis was conducted under 3 core IV assumptions: (1) relevance genetic variants are robustly associated with the exposure; (2) independence variants are not associated with confounders; and (3) exclusion restriction variants influence the outcome only through the exposure. BMI = body mass index, GWAS = genome-wide association studies, IV = instrumental variable, MR = Mendelian randomization, SNP = single nucleotide polymorphism.

### 2.2. Data sources

Genetic associations for seven adiposity traits were obtained from the UK Biobank, as curated by the Medical Research Council Integrative Epidemiology Unit (GWAS IDs: ukb-b-19953, ukb-b-8909, ukb-b-16407, ukb-b-20044, ukb-b-19393, ukb-b-17409, ukb-b-13354).^[12]^ These included BMI (N = 461,460), body fat percentage (N = 454,633), trunk fat percentage (N = 454,613), trunk fat mass (N = 454,588), whole-body fat mass (N = 454,137), trunk fat-free mass (N = 454,508), and whole-body fat-free mass (N = 454,850). Genetic associations for 5 hernia subtypes were sourced from the FinnGen consortium R8 release (GWAS IDs: finn-b-K11_DIAHER, finn-b-K11_FEMHER, finn-b-K11_HERING, finn-b-K11_UMBHER, finn-b-K11_VENTHER)^[13]^: diaphragmatic hernia (5741 cases, 190,557 controls), femoral hernia (700 cases, 190,557 controls), inguinal hernia (17,096 cases, 190,557 controls), umbilical hernia (4224 cases, 190,557 controls), and ventral hernia (3737 cases, 190,557 controls). All data sources are described in Table 1. Importantly, the exposure and outcome GWAS were derived from completely independent, non-overlapping cohorts (UK Biobank and FinnGen, respectively), thereby minimizing the risk of bias from sample overlap in our two-sample MR design.

**Table 1:** Characteristics of the GWAS data sources used for the MR analysis.

Phenotype	GWAS ID	GWAS population	Sample size	Cases/controls	N of SNPs	First author	Yr
Body mass index (BMI)	ukb-b-19,953	European	461,460	–	9,851,867	Ben Elsworth	2018
Body fat percentage	ukb-b-8909	European	454,633	–	9,851,867	Ben Elsworth	2018
Trunk fat percentage	ukb-b-16407	European	454,613	–	9,851,867	Ben Elsworth	2018
Trunk fat mass	ukb-b-20044	European	454,588	–	9,851,867	Ben Elsworth	2018
Whole body fat mass	ukb-b-19393	European	454,137	–	9,851,867	Ben Elsworth	2018
Trunk fat-free mass	ukb-b-17409	European	454,508	–	9,851,867	Ben Elsworth	2018
Whole body fat-free mass	ukb-b-13354	European	454,850	–	9,851,867	Ben Elsworth	2018
Diaphragmatic hernia	finn-b-K11_DIAHER	European	196,298	5741/190,557	16,380,395	FinnGen	2021
Femoral hernia	finn-b-K11_FEMHER	European	191,257	700/190,557	16,380,383	FinnGen	2021
Inguinal hernia	finn-b-K11_HERING	European	207,653	17096/190,557	16,380,436	FinnGen	2021
Umbilical hernia	finn-b-K11_UMBHER	European	194,781	4224/190,557	16,380,386	FinnGen	2021
Ventral hernia	finn-b-K11_VENTHER	European	194,294	3737/190,557	16,380,396	FinnGen	2021

BMI = body mass index, GWAS = genome-wide association study, MR = Mendelian randomization, SNP = single nucleotide polymorphism.

### 2.3. IV selection

For each exposure, we selected independent single-nucleotide polymorphism (SNPs) associated with the trait at genome-wide significance (*P* < 5 × 10^−8^). To ensure independence, SNPs were clumped using an LD threshold of R^2^ < 0.001 within a 10,000 kb window, using the European 1000 Genomes Project as the reference panel.^[14]^ We extracted the corresponding SNP-outcome effects from the FinnGen datasets. Palindromic SNPs with intermediate allele frequencies were harmonized to ensure effect alleles were consistent across exposure and outcome. The strength of each individual SNP was evaluated using the F‑statistic, which was calculated according to the formula: *F* = [*R*^2^ × (N − *k* − 1)]/[*k* × (1 − *R*^2^)], and SNPs with an *F*-statistic ≤ 10 were considered weak instruments and removed.^[15]^

### 2.4. MR analysis

For each exposure-outcome pair, we performed MR using three complementary methods with distinct underlying assumptions. IVW was selected as the primary method because it yields the most precise and statistically powerful causal estimate under the core assumption that all genetic instruments are valid (i.e., no horizontal pleiotropy), and represents the standard primary analytical approach in two-sample MR research.^[10]^ The MR Egger method was employed to allow for potential directional horizontal pleiotropy and provides consistent estimates under the Inside assumption, even if all instruments are invalid.^[16]^ The Weighted Median method can provide a consistent causal estimate if at least 50% of the weight in the analysis is derived from valid genetic instruments.^[17]^

### 2.5. Sensitivity analyses

For the significant exposure-outcome pairs identified in the primary IVW analysis, we conducted a series of sensitivity analyses to assess the robustness of our findings: Heterogeneity Analysis: Cochran’s *Q* test was used to assess heterogeneity among the SNP-specific causal estimates in the IVW and MR Egger methods.^[18]^ A significant *P*-value (<.05) indicated the presence of heterogeneity. If heterogeneity was present, we performed a random-effects IVW model to provide a more conservative estimate. Horizontal Pleiotropy: The intercept from MR Egger was used to test for directional horizontal pleiotropy.^[16]^ A *P*-value < .05 was considered evidence of pleiotropy. Exposures showing significant pleiotropy were flagged. Directionality Test: The Steiger test was performed to verify the causal direction (i.e., that the exposure causes the outcome, and not vice-versa).^[19]^ Exposure-outcome pairs failing this test were to be excluded. Visual Inspection and Sensitivity Plots: To graphically examine the distribution of effect estimates and recognize potentially influential variants, we constructed single-SNP forest plots, funnel plots, and scatter plots, together with leave-one-out analysis.^[20]^ and Outlier Removal and Confirmatory Analysis: To identify and correct for potentially biased SNP estimates, we applied the Radial IVW method.^[21]^ This method detects and removes influential outlier variants that disproportionately contribute to heterogeneity. After excluding these variants, we reperformed the MR analysis and reassessed heterogeneity. This approach allowed us to evaluate whether the primary findings remained consistent after removing potentially influential variants. All sensitivity and confirmatory analyses, including the Radial IVW approach for outlier removal, were prespecified as integral parts of our analytical pipeline to ensure the robustness of our findings.

### 2.6. Statistical analysis

All statistical analyses were performed using R software (version 4.4.1) with the TwoSampleMR and RadialMR packages.^[21,22]^ Effect sizes were reported as odds ratios (ORs) with corresponding 95% confidence intervals (CIs) per standard deviation increase in each continuous adiposity trait, representing the change in hernia risk associated with a genetically predicted increase of one standard deviation in the respective exposure. To account for multiple comparisons across the 5 hernia subtypes for a given exposure, we applied the Benjamini-Hochberg (BH) procedure to the unadjusted IVW *P*-values, yielding BH-adjusted *P*-values (*P*_BH_). An exposure-outcome pair was deemed statistically significant in the primary analysis if *P*_BH_ < .05. This correction was applied for each of the seven exposures independently.

## 3. Results

### 3.1. Characteristics of IVs

After clumping and harmonization, the number of SNPs used as IVs for each adiposity trait varied across the different outcomes, ranging from 306 to 463 (**Table S1**, Supplemental Digital Content 1). All selected SNPs exceeded the conventional threshold for weak instrument bias, with F-statistics well above 10 (minimum *F*-statistic > 29 for all exposures), indicating strong instruments and ensuring the reliability of the MR estimates.

### 3.2. Primary MR analysis

After BH correction, a consistent and divergent pattern of causal effects was the primary finding from our analysis. Across all 5 fat-mass-related adiposity measures (BMI, body fat percentage, trunk fat percentage, trunk fat mass, and whole-body fat mass), genetically predicted higher adiposity was robustly associated with a decreased risk of inguinal hernia and an increased risk of umbilical and ventral hernias. In contrast, no significant causal effects were observed for diaphragmatic or femoral hernias, or for any fat-free mass traits across any hernia subtype. The detailed results synthesized below are fully presented in Table S2, Supplemental Digital Content 2.

#### 3.2.1. Fat-mass adiposity traits and hernia risk

This consistent pattern is best illustrated by the results for BMI (Fig. 2). Genetically predicted higher BMI was significantly associated with a lower risk of inguinal hernia (IVW OR: 0.84, 95% CI: 0.76–0.92, *P*_BH_ < .001). In contrast, it was significantly associated with an increased risk of both umbilical hernia (IVW OR: 1.39, 95% CI: 1.18–1.63, *P*_BH_ < .001) and ventral hernia (IVW OR: 1.93, 95% CI: 1.65–2.26, *P*_BH_ < .001). No causal effects were detected for diaphragmatic hernia (IVW OR: 1.08, 95% CI: 0.94–1.25, *P*_BH_ = .347) or femoral hernia (IVW OR: 0.89, 95% CI: 0.62–1.28, *P*_BH_ = .526).

**Figure 2.**
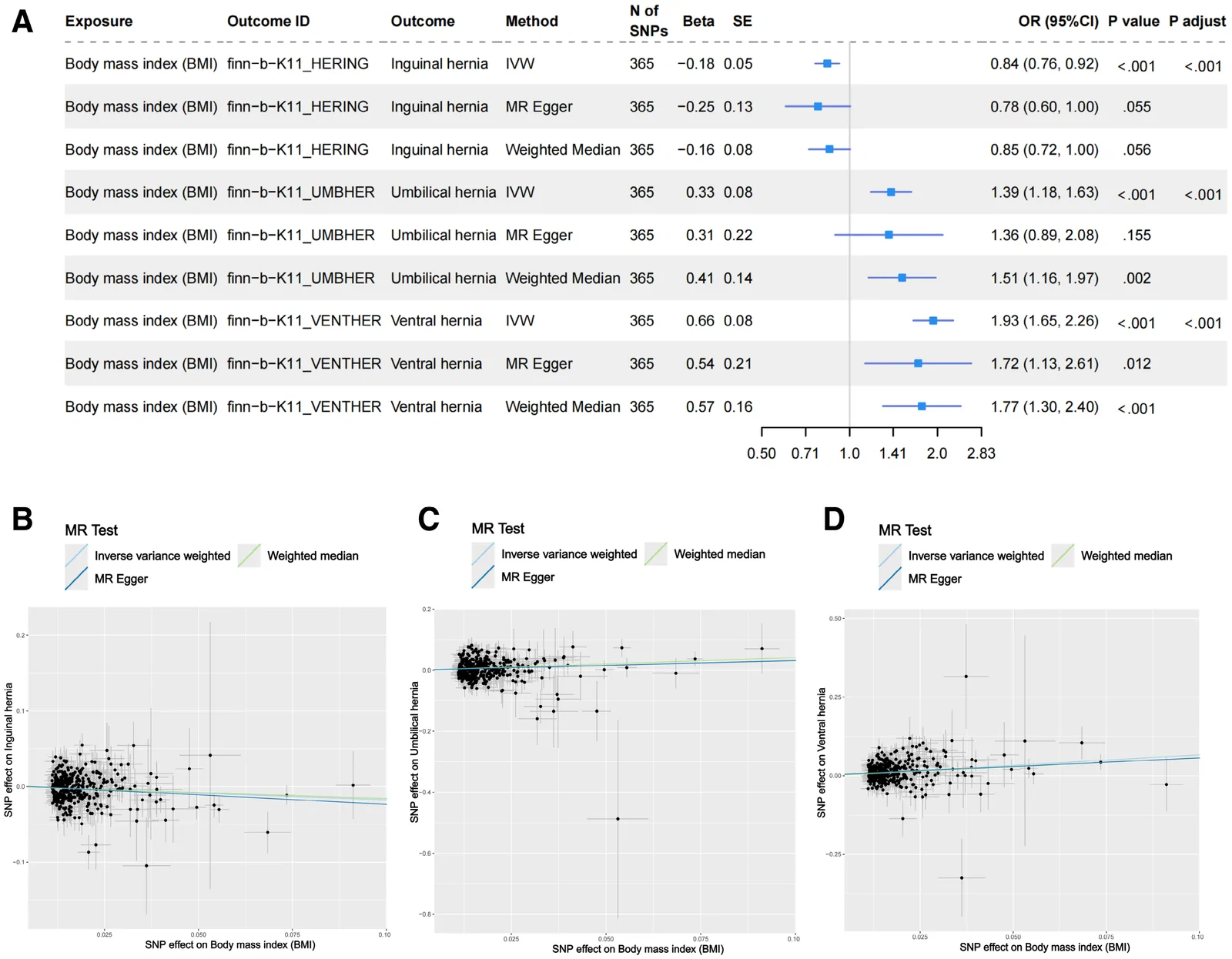
Primary Mendelian randomization estimates for body mass index on hernia subtypes. (A) Forest plot displaying the causal estimates (OR and 95% CI) from IVW, MR Egger, and weighted median methods for the association between genetically predicted BMI and each hernia subtype. (B–D) Scatter plots illustrating the relationship between SNP effects on BMI and on inguinal (B), umbilical (C), and ventral (D) hernias. The slopes of the lines represent the causal estimates from different MR methods. Higher genetically predicted BMI was associated with a lower risk of inguinal hernia but a higher risk of umbilical and ventral hernias. BMI = body mass index, CI = confidence interval, IVW = inverse-variance weighted, MR = Mendelian randomization, OR = odds ratio, SE = standard error, SNP = single nucleotide polymorphism.

This divergent pattern was highly consistent across all other fat-mass measures (body fat percentage, trunk fat percentage, trunk fat mass, and whole-body fat mass). As detailed in Table S2, Supplemental Digital Content 2 and Figures 3–6, all 4 traits replicated the protective effect for inguinal hernia and the risk-increasing effect for umbilical and ventral hernias, with IVW-derived *P*_BH_ < .05. The magnitude of these effects was comparable across traits; for example, the OR for ventral hernia ranged from 1.77 (whole-body fat mass) to 2.50 (body fat percentage). The MR Egger and weighted median methods provided estimates that were directionally consistent with the IVW findings for all significant outcomes.

**Figure 3.**
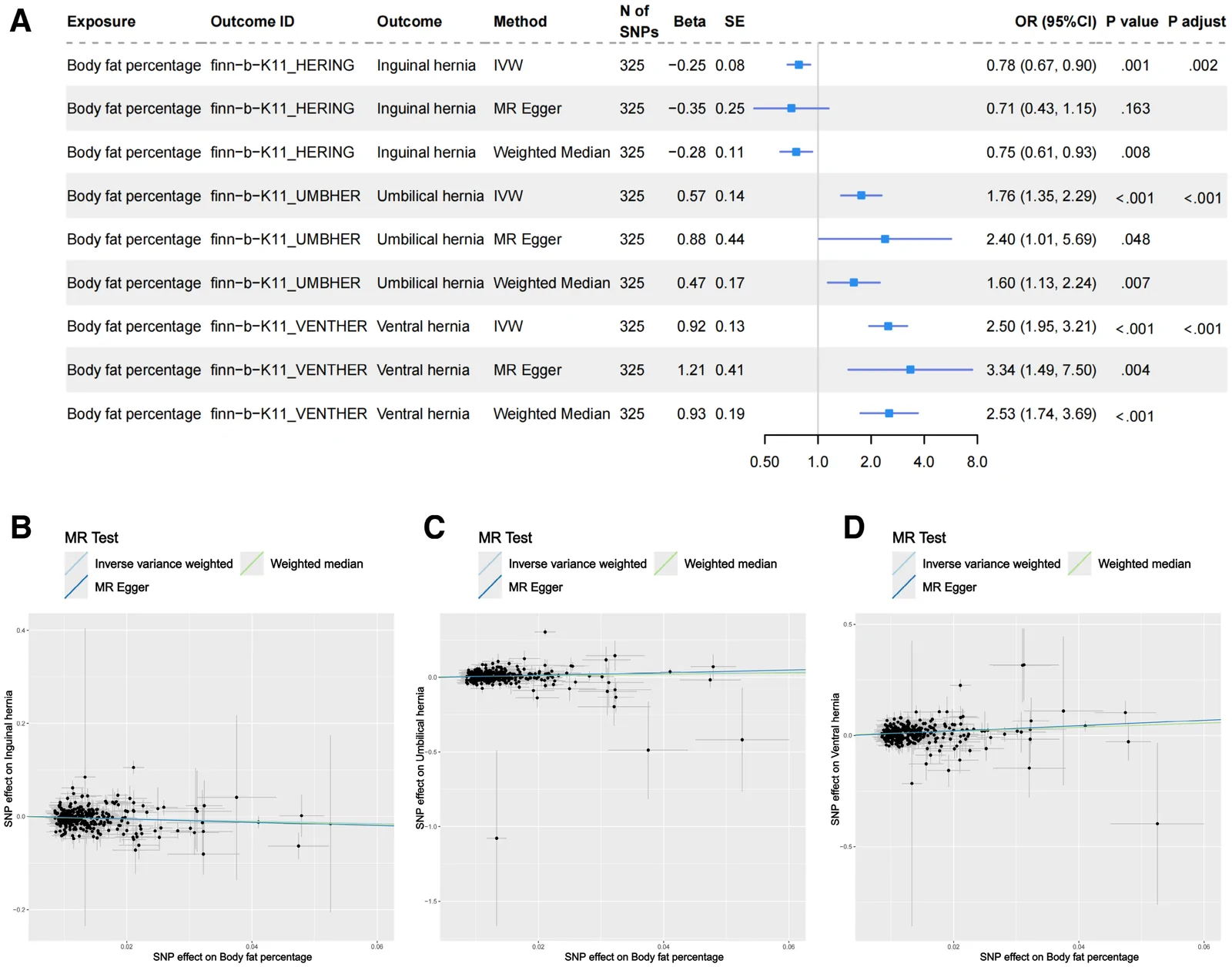
Primary Mendelian randomization estimates for body fat percentage on hernia subtypes. (A) Forest plot of causal estimates (OR and 95% CI) from IVW, MR Egger, and weighted median methods for body fat percentage on each hernia subtype. (B–D) Scatter plots of SNP effects on body fat percentage versus inguinal (B), umbilical (C), and ventral (D) hernias. The patterns mirror those observed for BMI: higher body fat percentage was associated with a lower inguinal hernia risk and higher umbilical and ventral hernia risks. BMI = body mass index, CI = confidence interval, IVW = inverse-variance weighted, MR = Mendelian randomization, OR = odds ratio, SE = standard error, SNP = single nucleotide polymorphism.

**Figure 4.**
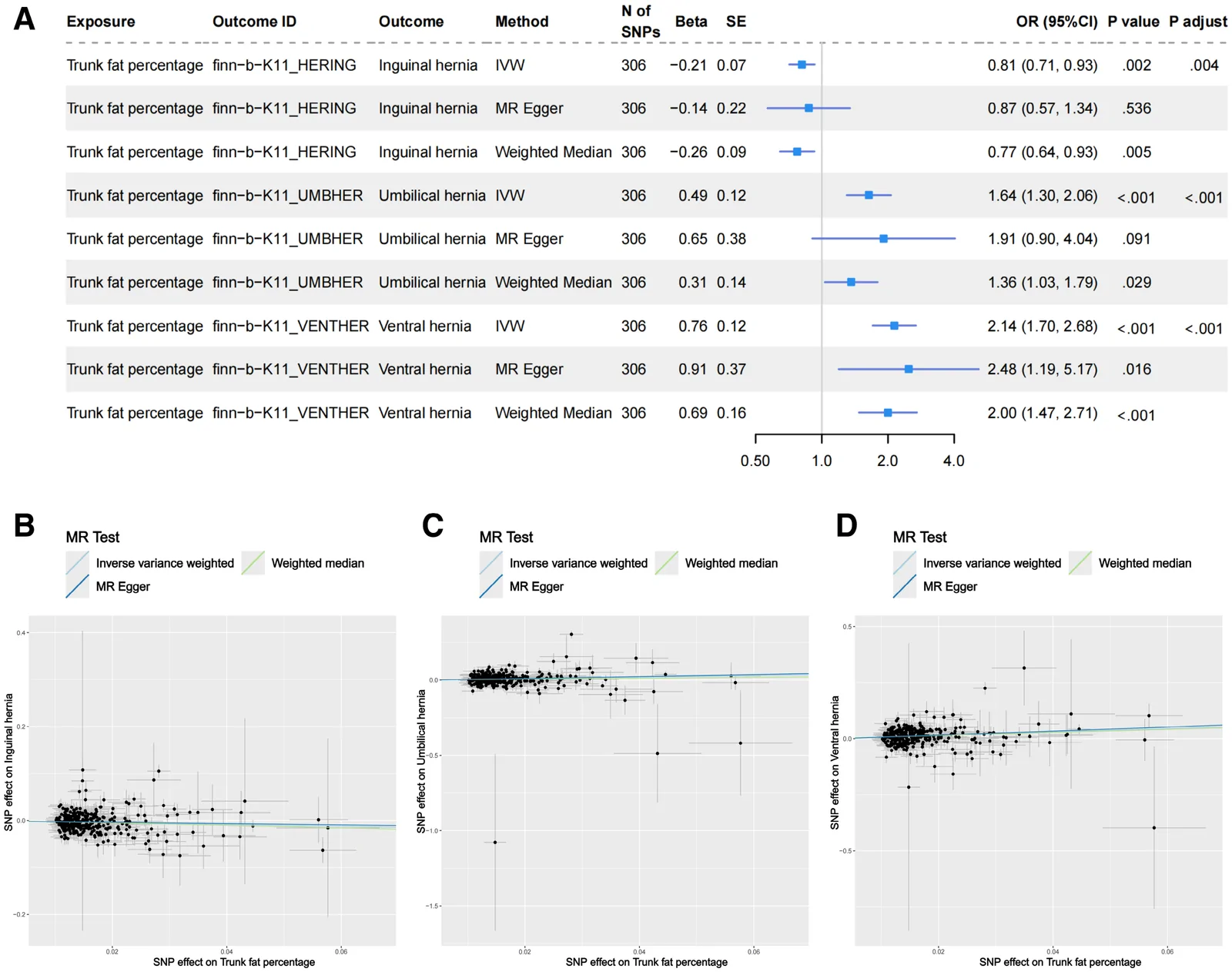
Primary Mendelian randomization estimates for trunk fat percentage on hernia subtypes. (A) Forest plot of causal estimates (OR and 95% CI) from IVW, MR Egger, and weighted median methods for trunk fat percentage on each hernia subtype. (B–D) Scatter plots of SNP effects on trunk fat percentage versus inguinal (B), umbilical (C), and ventral (D) hernias. Trunk fat percentage, as a measure of central adiposity, showed the same divergent association pattern as overall adiposity measures. CI = confidence interval, IVW = inverse-variance weighted, MR = Mendelian randomization, OR = odds ratio, SE = standard error, SNP = single nucleotide polymorphism.

**Figure 5.**
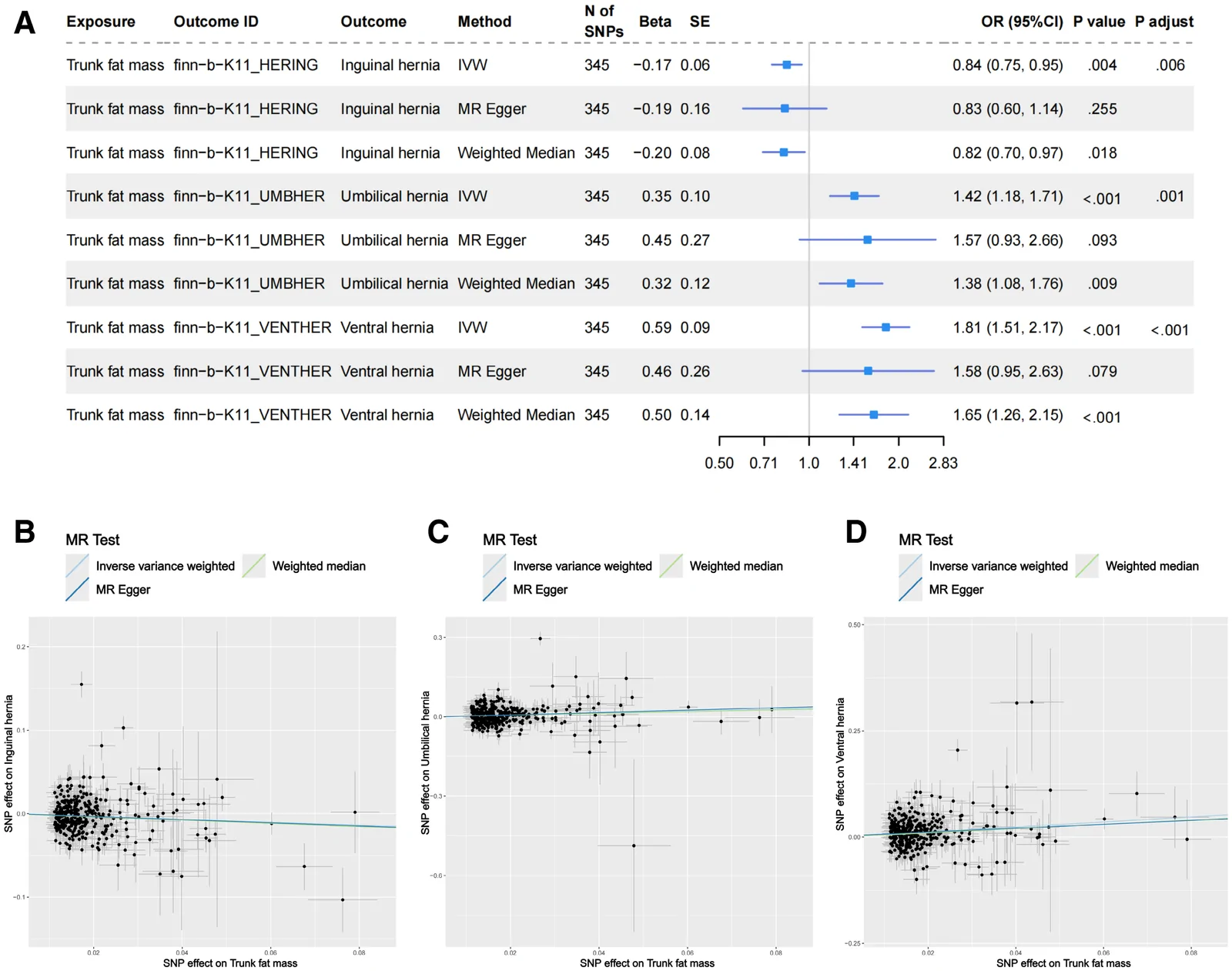
Primary Mendelian randomization estimates for trunk fat mass on hernia subtypes. (A) Forest plot of causal estimates (OR and 95% CI) from IVW, MR Egger, and weighted median methods for trunk fat mass on each hernia subtype. (B–D) Scatter plots of SNP effects on trunk fat mass versus inguinal (B), umbilical (C), and ventral (D) hernias. The results are consistent with those for trunk fat percentage, further supporting the role of central adiposity. CI = confidence interval, IVW = inverse-variance weighted, MR = Mendelian randomization, OR = odds ratio, SE = standard error, SNP = single nucleotide polymorphism.

**Figure 6.**
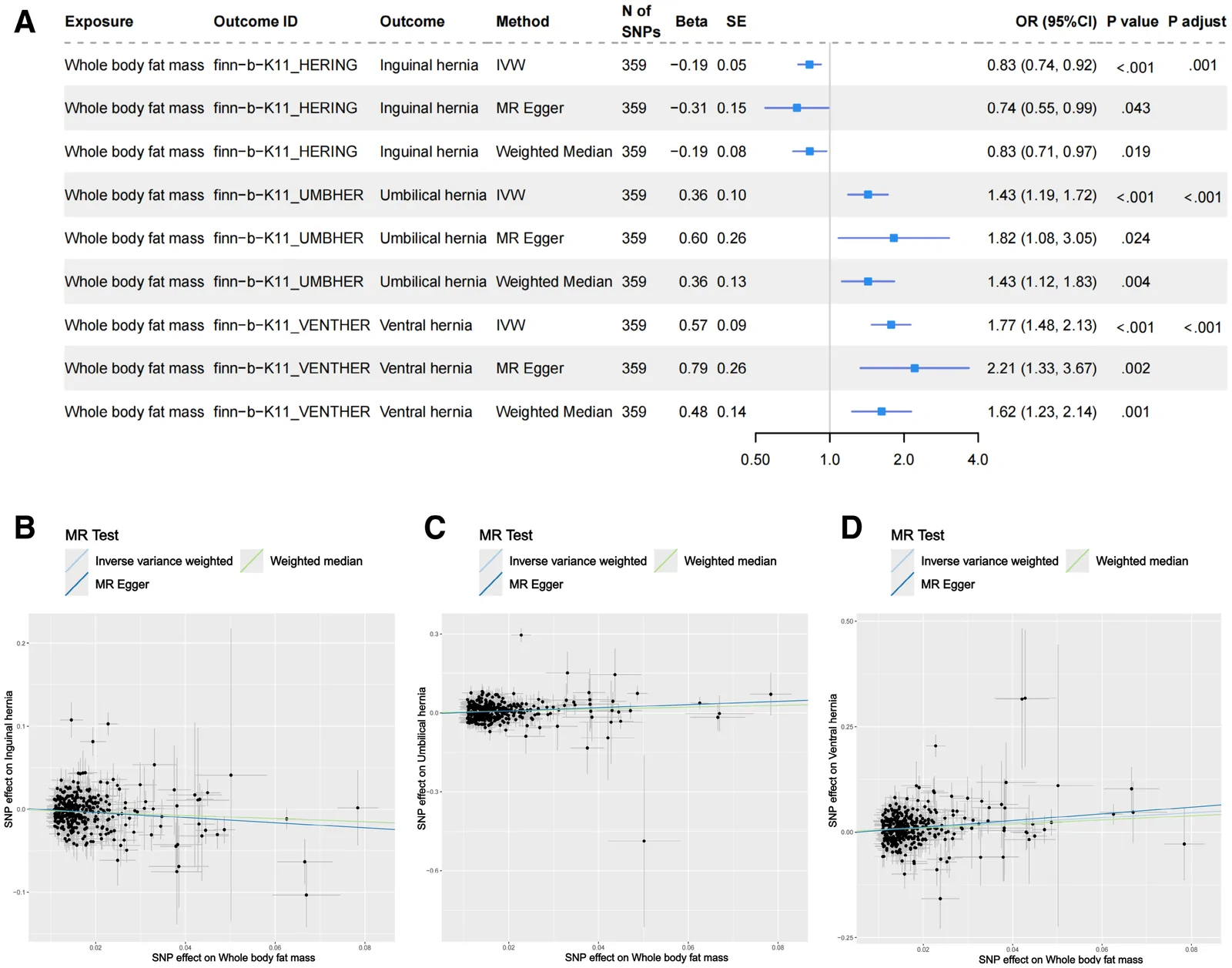
Primary Mendelian randomization estimates for whole-body fat mass on hernia subtypes. (A) Forest plot of causal estimates (OR and 95% CI) from IVW, MR Egger, and weighted median methods for whole-body fat mass on each hernia subtype. (B–D) Scatter plots of SNP effects on whole body fat mass versus inguinal (B), umbilical (C), and ventral (D) hernias. The findings replicate the divergent pattern observed with BMI and other adiposity measures. CI = confidence interval, IVW = inverse-variance weighted, MR = Mendelian randomization, OR = odds ratio, SE = standard error, SNP = single nucleotide polymorphism.

#### 3.2.2. Fat-free mass traits and hernia risk

In contrast to the fat mass measures, genetically predicted higher fat-free mass (both trunk and whole body) showed no significant causal association with any of the 5 hernia subtypes after multiple testing correction. All P_BH_ values for the IVW estimates were >.05, with ORs all close to the null value of 1 (Table S2, Supplemental Digital Content 2). For example, the IVW estimates for trunk fat-free mass on inguinal hernia (OR: 0.92, 95% CI: 0.80–1.05, *P*_BH_ = .321) and ventral hernia (OR: 1.13, 95% CI: 0.91–1.40, *P*_BH_ = .321) were not statistically significant.

### 3.3. Sensitivity analyses

Sensitivity analyses were conducted for the 15 exposure-outcome pairs (5 fat mass traits × 3 significant hernia outcomes) that showed significant associations in the primary analysis. The results for the 2 fat-free mass traits, which were null, were not carried forward for further sensitivity testing. Forest plots depicting the causal estimates of individual SNPs for each significant pair are provided in Figure S1, Supplemental Digital Content 3. Cochran’s *Q* test revealed significant heterogeneity (*P* < .05) for most of these 15 significant pairs, as shown in Table S3, Supplemental Digital Content 4. For instance, the analysis of body fat percentage on inguinal hernia showed a *Q P*-value of 2.60 × 10^−11^, with an *I*^2^ of 37.7%. Funnel plots (Fig. 7) were also inspected and confirmed the presence of heterogeneity. This heterogeneity justified the use of a random-effects IVW model in the confirmatory stage. Critically, the MR Egger intercept test did not identify statistically significant evidence of directional horizontal pleiotropy for any of these pairs (all *P* > .05), suggesting that pleiotropy is unlikely to have systematically biased the IVW estimates (Table S4, Supplemental Digital Content 5). The Steiger test confirmed the correct causal direction for all significant pairs, with the exposure explaining significantly more variance in the outcome than vice-versa (all *P* < 2.2 × 10^−16^) (Table S5, Supplemental Digital Content 6). This confirms that reverse causation is unlikely to explain our findings. Leave-one-out sensitivity analyses (Figure S2, Supplemental Digital Content 7) demonstrated that no single SNP was disproportionately driving the overall causal estimates, providing further reassurance that the findings are not artifacts of a few outlying variants.

**Figure 7.**
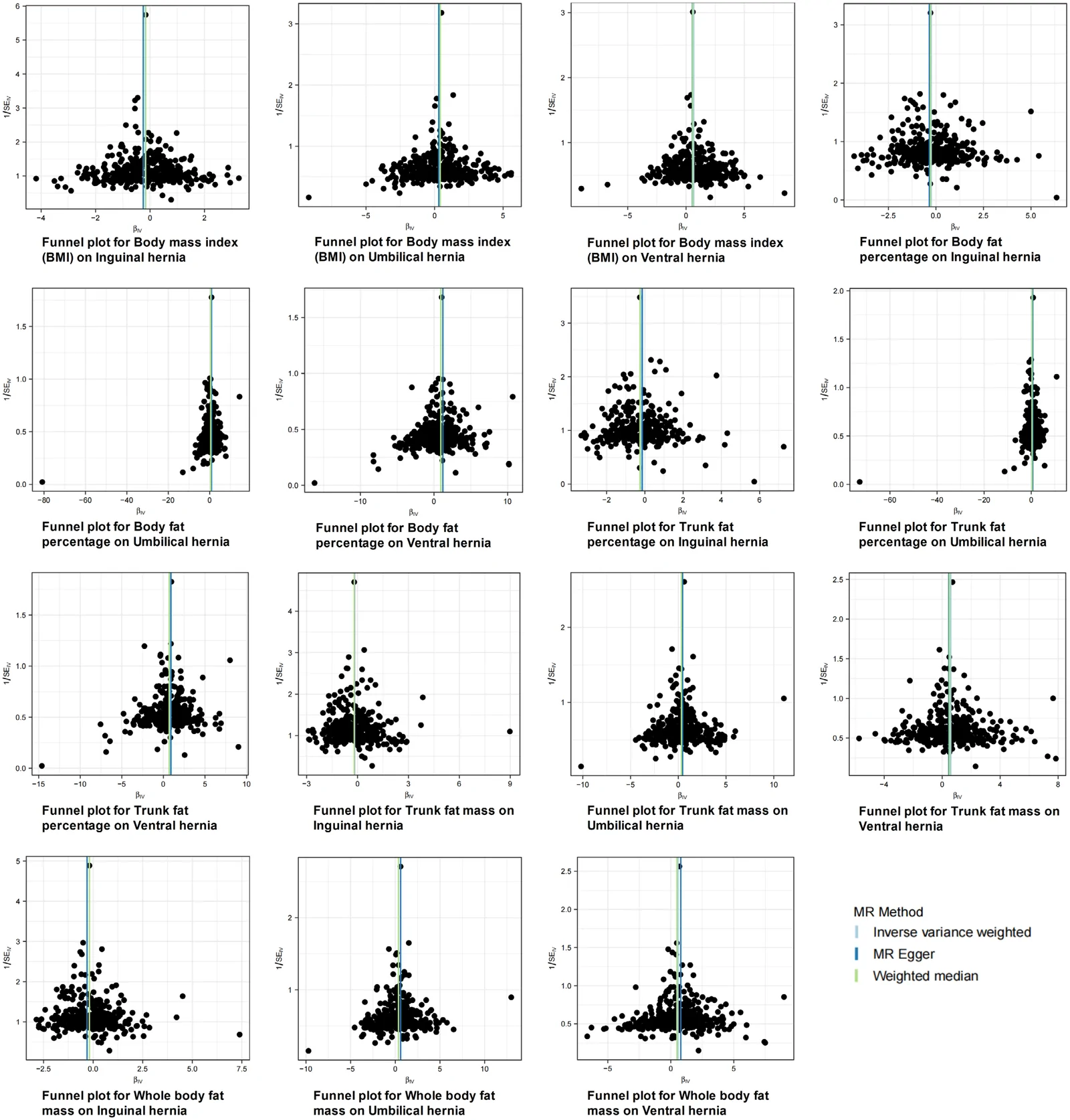
Funnel plots for heterogeneity assessment. Funnel plots showing the distribution of SNP‑specific causal estimates (x-axis) against their precision (1/SE, y-axis) for the 15 significant exposure–outcome pairs (five fat-mass traits × three hernia subtypes). In the absence of heterogeneity, points should symmetrically funnel around the overall estimate. Asymmetry or spread beyond the expected funnel shape indicates heterogeneity, which was observed for most pairs prior to outlier removal. BMI = body mass index, IV = instrumental variable, MR = Mendelian randomization, SE = standard error, SNP = single nucleotide polymorphism.

### 3.4. Confirmatory MR analysis after outlier removal

To address the observed heterogeneity, we performed a Radial IVW analysis to identify and remove outlier SNPs (Table S6, Supplemental Digital Content 8). The radial plots depicting the detection of outlier variants are provided in Figure 8. The final set of IVs retained after outlier removal for each exposure–outcome pair is summarized in Table S7, Supplemental Digital Content 9. The confirmatory MR results after this process are detailed in Table S8, Supplemental Digital Content 10. Forest plots summarizing the confirmatory estimates for each significant exposure-outcome pair are presented in Figure S3–S7, Supplemental Digital Content 11. The removal of outliers strengthened the primary findings and successfully eliminated heterogeneity. For example, the effect of BMI on inguinal hernia remained protective (Radial-IVW OR: 0.84, 95% CI: 0.77–0.92, *P*_BH_ < .001), with heterogeneity becoming non-significant (*P*-heterogeneity = .99). The positive associations with umbilical hernia (e.g., BMI OR: 1.34, 95% CI: 1.15–1.56, *P*_BH_ < .001) and ventral hernia (e.g., BMI OR: 1.77, 95% CI: 1.51–2.08, *P*_BH_ < .001) were also robust and free from significant heterogeneity after outlier removal. These confirmatory estimates were highly consistent across all 5 fat-mass adiposity traits, reinforcing the reliability of the initial findings.

**Figure 8.**
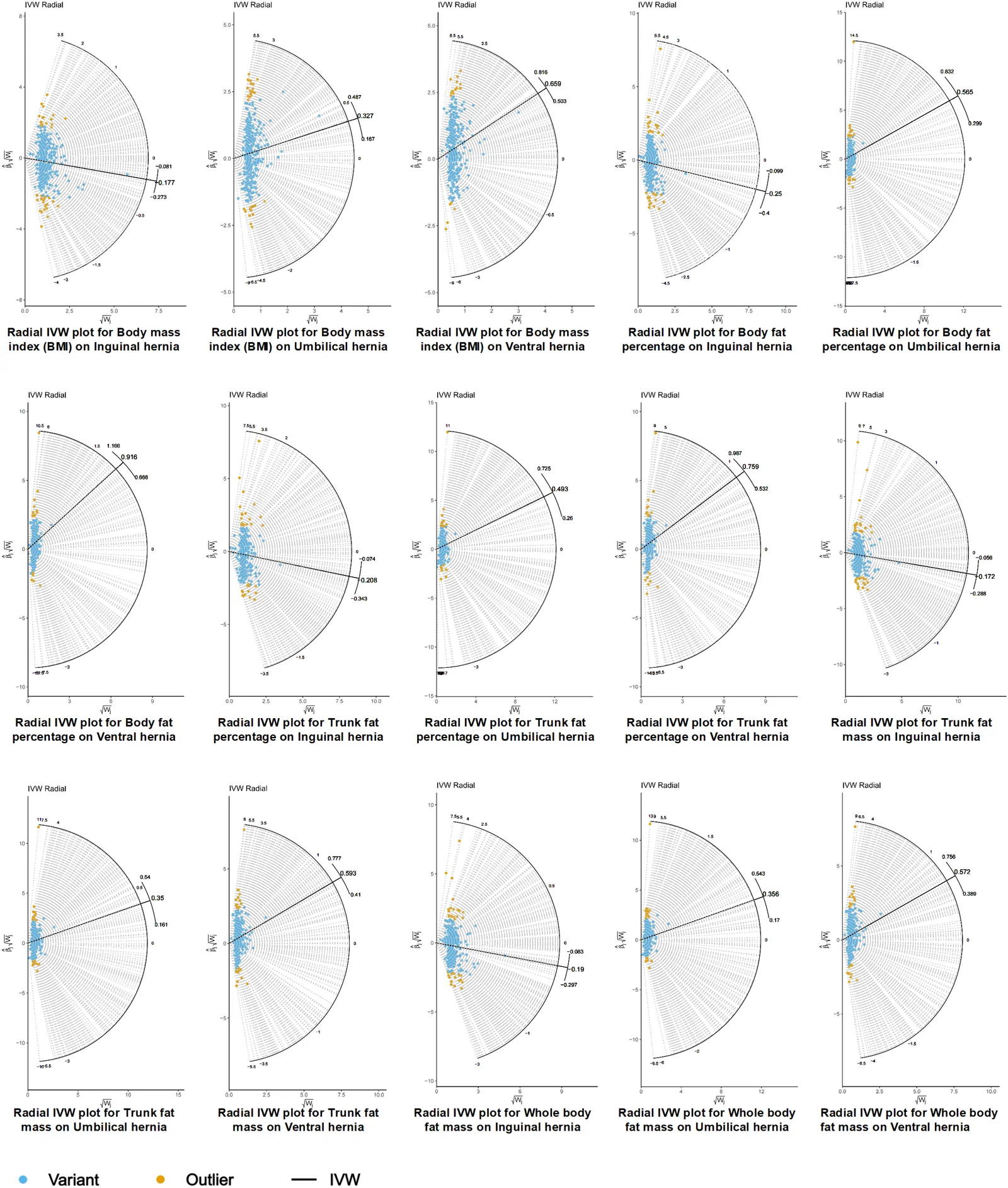
Radial IVW plots for outlier detection. Radial plots from the Radial IVW analysis, used to identify outlier SNPs that disproportionately contribute to heterogeneity. Each panel represents a significant exposure–outcome pair. Each point represents an IV, with its distance from the origin proportional to the IV’s contribution to the overall *Q* statistic. Outliers (highlighted in orange) were detected and removed prior to confirmatory MR analysis to eliminate heterogeneity and obtain more stable estimates. BMI = body mass index, IV = instrumental variable, IVW = inverse-variance weighted, MR = Mendelian randomization, SNP = single nucleotide polymorphism.

## 4. Discussion

In this comprehensive two-sample MR study, we provide suggestive causal evidence that adiposity may exert divergent effects on the etiology of different hernia subtypes. We observed that genetically predicted higher general and central adiposity was associated with a lower risk of inguinal hernia but a higher risk of umbilical and ventral hernias. No significant associations were detected for diaphragmatic or femoral hernias. These patterns were consistent across multiple adiposity indicators, supported by various sensitivity analyses, and remained stable after outlier adjustment.

### 4.1. Divergent effects on hernia subtypes

The most striking finding is the opposing direction of association for inguinal versus abdominal (umbilical and ventral) hernias. The protective MR-based association of higher BMI and body fat with inguinal hernia risk is a counterintuitive but not unprecedented observation.^[4]^ A potential biological mechanism is the “fat plug” hypothesis.^[23]^ It suggests that in the inguinal canal, a protrusion of preperitoneal fat can occur without a true peritoneal sac, which may be clinically misclassified or may physically obstruct the canal, preventing the descent of a proper hernia sac containing abdominal viscera.^[9,24]^ Furthermore, individuals with higher adiposity may have a different collagen structure or increased connective tissue deposition, potentially strengthening the inguinal ring.^[9]^ This finding has clinical implications, suggesting that relying on a uniform preventative strategy of weight loss for all hernia types may be overly simplistic.^[25,26]^ The data, which reflect lifelong exposure to higher adiposity, suggest that a genetically lower BMI is associated with a higher risk of inguinal hernia. However, the direct effect of intentional weight loss in adulthood on inguinal hernia risk cannot be inferred from this study and warrants cautious interpretation. Prospective studies are needed to understand the net clinical impact of weight change on the full spectrum of hernia risk.

Conversely, the increased risk for umbilical and ventral hernias aligns with the traditional mechanical theory of hernia formation. Chronically elevated IAP, a direct consequence of central obesity, places continuous stress on the abdominal wall.^[27–29]^ This is particularly relevant for the midline, which includes the umbilicus (a congenital weak point) and areas of previous surgical incisions (for ventral hernias).^[30,31]^ The consistent results across trunk-specific fat measures (trunk fat percentage and mass) further support the role of central adiposity and elevated IAP in driving these abdominal wall hernias.

### 4.2. Comparison with previous literature

The relationship between obesity and hernia risk has been debated, with observational studies yielding conflicting results: some implicating obesity as a risk factor for various hernia types,^[5]^ others reporting an inverse association with inguinal hernia specifically.^[4,32,33]^ These inconsistencies likely stem from residual confounding and reverse causation inherent to observational designs.^[8]^ Obesity is closely linked to physical inactivity, dietary patterns, and comorbidities such as chronic cough or constipation: factors that independently influence hernia risk,^[34–37]^ making it difficult to isolate the causal effect of adiposity using conventional approaches.

MR, which uses genetic variants randomly allocated at conception, helps mitigate these limitations.^[10,38]^ Although MR reduces confounding and reverse causation, residual bias: particularly unmeasured horizontal pleiotropy: cannot be entirely excluded, even after comprehensive sensitivity analyses. Our results indicate that genetically predicted higher adiposity is associated with a lower risk of inguinal hernia. These findings are supported by an increasing number of MR studies conducted in European populations.^[24,25,39]^ Collectively, such evidence illustrates the value of MR in clarifying causal relationships that may be masked in traditional observational research.^[40]^

The increased risk of umbilical and ventral hernias observed in this study aligns with the classic pathophysiological mechanism and is consistent with MR findings in other IAP-related conditions, such as gastroesophageal reflux disease.^[41]^

### 4.3. Strengths and limitations

This study has several strengths. First, the use of two-sample MR with large, well-powered GWAS datasets (UK Biobank and FinnGen) provides high statistical power and precision.^[13,42]^ Second, we examined multiple adiposity measures, allowing us to assess both general and central obesity. Third, the comprehensive sensitivity analyses, including tests for pleiotropy, heterogeneity, and directionality, alongside an outlier-robust confirmatory analysis, ensure the robustness of our findings.^[16,17,21]^

Nevertheless, several limitations should be acknowledged with their potential impacts on interpretation. First, horizontal pleiotropy cannot be fully excluded. If present, it may bias causal estimates toward or away from the null, although non‑significant MR-Egger intercepts alleviate this concern.^[10,43]^ Second, the study was restricted to European ancestry, which may limit generalizability to other ethnic populations with distinct genetic backgrounds and hernia epidemiology.^[44,45]^ Third, phenotype misclassification in biobank data may occur, which would most likely bias results toward the null and lead to underestimation of true effects.^[46]^ In addition, hernia outcomes derived from biobank or registry-based definitions may vary in diagnostic accuracy and clinical severity across subtypes, which could influence subtype-specific effect estimates. Finally, this study reflects lifelong genetic predisposition to adiposity, which may not fully represent the effect of weight change during adulthood, thereby limiting direct translation to clinical weight management advice.^[47,48]^

## 5. Conclusions

This MR study suggests that adiposity may exert divergent, potentially causal associations with hernia subtypes in European populations. Genetically predicted higher adiposity is associated with a reduced risk of inguinal hernia but an increased risk of umbilical and ventral hernias. These findings advance our understanding of hernia pathophysiology and support the need for tailored clinical risk stratification and prevention strategies according to hernia type. Future studies are warranted to validate these results in diverse ancestral populations and to clarify the precise biological mechanisms underlying these divergent effects, particularly the protective mechanism in the inguinal region.

## Acknowledgements

We gratefully acknowledge the FinnGen consortium and the IEU OpenGWAS database for providing publicly accessible genome-wide association study summary statistics. We also thank all investigators and participants of the original studies for their valuable contributions.

## Author contributions

**Conceptualization:** Yao Lu, Dechun Liu.

**Data curation:** Yao Lu, Huiping Yuan.

**Methodology:** Yao Lu, Huiping Yuan.

**Software:** Yao Lu, Huiping Yuan.

**Validation:** Yao Lu, Ke Zhang.

**Supervision:** Ke Zhang, Dechun Liu.

**Writing – review & editing:** Ke Zhang, Dechun Liu.

**Writing – original draft:** Yao Lu, Huiping Yuan.































## References

[1] Fitzgibbons RJ Forse RA. Groin hernias in adults. N Engl J Med. 2015;372:756–63. 10.1056/NEJMcp1404068 25693015

[2] Poulose BK Shelton J Phillips S. Epidemiology and cost of ventral hernia repair: making the case for hernia research. Hernia. 2012;16:179–83. 10.1007/s10029-011-0879-9 21904861

[3] Kingsnorth A LeBlanc K. Hernias: inguinal and incisional. Lancet (London, England). 2003;362:1561–71. 10.1016/S0140-6736(03)14746-0 14615114

[4] Rosemar A Angerås U Rosengren A. Body mass index and groin hernia: a 34-year follow-up study in Swedish men. Ann Surg. 2008;247:1064–8. 10.1097/SLA.0b013e31816b4399 18520236

[5] Lau B Kim H Haigh PI Tejirian T. Obesity increases the odds of acquiring and incarcerating noninguinal abdominal wall hernias. Am Surg. 2012;78:1118–21. 23025954

[6] Sugerman HJ DeMaria EJ Felton WL Nakatsuka M Sismanis A. Increased intra-abdominal pressure and cardiac filling pressures in obesity-associated pseudotumor cerebri. Neurology. 1997;49:507–11. 10.1212/wnl.49.2.507 9270586

[7] Henriksen NA Mortensen JH Sorensen LT. The collagen turnover profile is altered in patients with inguinal and incisional hernia. Surgery. 2015;157:312–21. 10.1016/j.surg.2014.09.006 25616945

[8] Davey Smith G Hemani G. Mendelian randomization: genetic anchors for causal inference in epidemiological studies. Hum Mol Genet. 2014;23:R89–98. 10.1093/hmg/ddu328 PMC4170722 25064373

[9] Rosca R Paduraru DN Bolocan A Musat F Ion D Andronic O. A comprehensive review of inguinal hernia occurrence in obese individuals. Maedica (Bucur). 2023;18:692–8. 10.26574/maedica.2023.18.4.692 PMC10859212 38348082

[10] Burgess S Butterworth A Thompson SG. Mendelian randomization analysis with multiple genetic variants using summarized data. Genet Epidemiol. 2013;37:658–65. 10.1002/gepi.21758 PMC4377079 24114802

[11] Burgess S Small DS Thompson SG. A review of instrumental variable estimators for Mendelian randomization. Stat Methods Med Res. 2017;26:2333–55. 10.1177/0962280215597579 PMC5642006 26282889

[12] Elsworth B Lyon M Alexander T. The MRC IEU OpenGWAS data infrastructure. 2020.

[13] Kurki MI Karjalainen J Palta P. FinnGen provides genetic insights from a well-phenotyped isolated population. Nature. 2023;613:508–18. 10.1038/s41586-022-05473-8 PMC9849126 36653562

[14] Auton A Brooks LD Durbin RM; 1000 Genomes Project Consortium. A global reference for human genetic variation. Nature. 2015;526:68–74. 10.1038/nature15393 PMC4750478 26432245

[15] Burgess S Thompson SG. Improving bias and coverage in instrumental variable analysis with weak instruments for continuous and binary outcomes. Stat Med. 2012;31:1582–600. 10.1002/sim.4498 22374818

[16] Bowden J Davey Smith G Burgess S. Mendelian randomization with invalid instruments: effect estimation and bias detection through Egger regression. Int J Epidemiol. 2015;44:512–25. 10.1093/ije/dyv080 PMC4469799 26050253

[17] Bowden J Davey Smith G Haycock PC Burgess S. Consistent estimation in mendelian randomization with some invalid instruments using a weighted median estimator. Genet Epidemiol. 2016;40:304–14. 10.1002/gepi.21965 PMC4849733 27061298

[18] Greco MFD Minelli C Sheehan NA Thompson JR. Detecting pleiotropy in Mendelian randomisation studies with summary data and a continuous outcome. Stat Med. 2015;34:2926–40. 10.1002/sim.6522 25950993

[19] Hemani G Tilling K Davey Smith G. Orienting the causal relationship between imprecisely measured traits using GWAS summary data. PLoS Genet. 2017;13:e1007081. 10.1371/journal.pgen.1007081 PMC5711033 29149188

[20] Burgess S Bowden J Fall T Ingelsson E Thompson SG. Sensitivity analyses for robust causal inference from Mendelian randomization analyses with multiple genetic variants. Epidemiology. 2017;28:30–42. 10.1097/EDE.0000000000000559 PMC5133381 27749700

[21] Bowden J Spiller W Del Greco M F. Improving the visualization, interpretation and analysis of two-sample summary data Mendelian randomization via the radial plot and radial regression. Int J Epidemiol. 2018;47:1264–78. 10.1093/ije/dyy101 PMC6124632 29961852

[22] Hemani G Zheng J Elsworth B. The MR-Base platform supports systematic causal inference across the human phenome. Elife. 2018;7:e34408. 10.7554/eLife.34408 PMC5976434 29846171

[23] Callaghan CJ Whitaker RH. Obturator canal fat plug: a pre-hernial condition? Clin Anat. 2002;15:276–9. 10.1002/ca.10025 12112355

[24] Hemberg A Montgomery A Holmberg H Nordin P. Waist circumference is not superior to body mass index in predicting groin hernia repair in either men or women. World J Surg. 2022;46:401–8. 10.1007/s00268-021-06359-y PMC8724106 34727205

[25] Shi H Peng X Lin Y. Association between different obesity metrics and risk of inguinal hernia. Updates Surg. 2025;77:567–74. 10.1007/s13304-025-02062-y 39821601

[26] Ravanbakhsh S Batech M Tejirian T. Increasing body mass index is inversely related to groin hernias. Am Surg. 2015;81:1043–6. 26463305

[27] Cobb WS Burns JM Kercher KW Matthews BD James Norton H Todd Heniford B. Normal intraabdominal pressure in healthy adults. J Surg Res. 2005;129:231–5. 10.1016/j.jss.2005.06.015 16140336

[28] Lee YY McColl KEL. Disruption of the gastroesophageal junction by central obesity and waist belt: role of raised intra-abdominal pressure. Dis Esophagus. 2015;28:318–25. 10.1111/dote.12202 24575877

[29] Muresan M Muresan S Bara T. The intraabdominal pressure A real indicator of the tension free principle during anterior wall repair procedure after incisional hernias. Ann Ital Chir. 2015;86:421–6. 26418543

[30] De Santis L Frigo F Bruttocao A Terranova O. Pathophysiology of giant incisional hernias with loss of abdominal wall substance. Acta Biomed. 2003;74:34–7. 15055031

[31] Hegazy AA. Anatomy and embryology of umbilicus in newborns: a review and clinical correlations. Front Med. 2016;10:271–7. 10.1007/s11684-016-0457-8 27473223

[32] Ruhl CE Everhart JE. Risk factors for inguinal hernia among adults in the US population. Am J Epidemiol. 2007;165:1154–61. 10.1093/aje/kwm011 17374852

[33] De Goede B Timmermans L Van Kempen BJH. Risk factors for inguinal hernia in middle-aged and elderly men: results from the Rotterdam Study. Surgery. 2015;157:540–6. 10.1016/j.surg.2014.09.029 25596770

[34] Landt EM Çolak Y Nordestgaard BG Lange P Dahl M. Risk and impact of chronic cough in obese individuals from the general population. Thorax. 2022;77:223–30. 10.1136/thoraxjnl-2020-216351 34230095

[35] Yurtdaş G Acar-Tek N Akbulut G. Risk factors for constipation in adults: a cross-sectional study. J Am Coll Nutr. 2020;39:713–9. 10.1080/07315724.2020.1727380 32077808

[36] Ashindoitiang JA Ibrahim NA Akinlolu OO. Risk factors for inguinal hernia in adult male Nigerians: a case control study. Int J Surg. 2012;10:364–7. 10.1016/j.ijsu.2012.05.016 22676975

[37] Kartal A Yalcin M Citgez B Uzunkoy A. The effect of chronic constipation on the development of inguinal herniation. Hernia. 2017;21:531–5. 10.1007/s10029-017-1604-0 28393306

[38] Skrivankova VW Richmond RC Woolf BAR. Strengthening the reporting of observational studies in epidemiology using Mendelian randomization: the STROBE-MR statement. JAMA. 2021;326:1614–21. 10.1001/jama.2021.18236 34698778

[39] Li Y Li B Meng F. Evaluating the relationship between standing height, body mass index, body fat percentage with risk of inguinal hernia: a Mendelian randomization study. Sci Rep. 2024;14:26402. 10.1038/s41598-024-78122-x PMC11531599 39488648

[40] Sheehan NA Didelez V Burton PR Tobin MD. Mendelian randomisation and causal inference in observational epidemiology. PLoS Med. 2008;5:e177. 10.1371/journal.pmed.0050177 PMC2522255 18752343

[41] Green HD Beaumont RN Wood AR. Genetic evidence that higher central adiposity causes gastro-oesophageal reflux disease: a Mendelian randomization study. Int J Epidemiol. 2020;49:1270–81. 10.1093/ije/dyaa082 PMC7750946 32588049

[42] Bycroft C Freeman C Petkova D. The UK Biobank resource with deep phenotyping and genomic data. Nature. 2018;562:203–9. 10.1038/s41586-018-0579-z PMC6786975 30305743

[43] Hemani G Bowden J Davey Smith G. Evaluating the potential role of pleiotropy in Mendelian randomization studies. Hum Mol Genet. 2018;27:R195–208. 10.1093/hmg/ddy163 PMC6061876 29771313

[44] Martin AR Kanai M Kamatani Y Okada Y Neale BM Daly MJ. Clinical use of current polygenic risk scores may exacerbate health disparities. Nat Genet. 2019;51:584–91. 10.1038/s41588-019-0379-x PMC6563838 30926966

[45] Sirugo G Williams SM Tishkoff SA. The missing diversity in human genetic studies. Cell. 2019;177:26–31. 10.1016/j.cell.2019.02.048 PMC7380073 30901543

[46] Zelicha H Bell DS Chen Y Livingston EH. Potential for error when relying on administrative data. Br J Surg. 2025;112:znaf139. 10.1093/bjs/znaf139 PMC12268322 40673449

[47] Burgess S Timpson NJ Ebrahim S Davey Smith G. Mendelian randomization: where are we now and where are we going? Int J Epidemiol. 2015;44:379–88. 10.1093/ije/dyv108 26085674

[48] Holmes MV Ala-Korpela M Smith GD. Mendelian randomization in cardiometabolic disease: challenges in evaluating causality. Nat Rev Cardiol. 2017;14:577–90. 10.1038/nrcardio.2017.78 PMC5600813 28569269

