# Supplementary material for: Divergent associations between adiposity and hernia subtypes in European populations: A Mendelian randomization study
Source: Medicine (Baltimore). 2026 Oct 16;105(42):e51209. doi: 10.1097/MD.0000000000051209 (PMC13681110; doi:10.1097/MD.0000000000051209)
Supplement: Supplementary file 1 [file medi-105-e51209-s003.pdf]

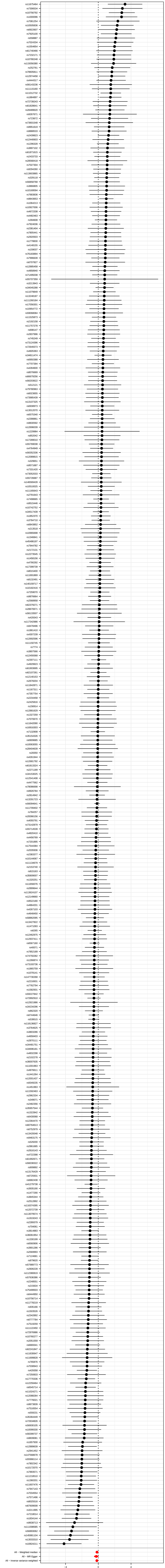

MR effect size for Body mass index (BMI) on Inguinal hernia

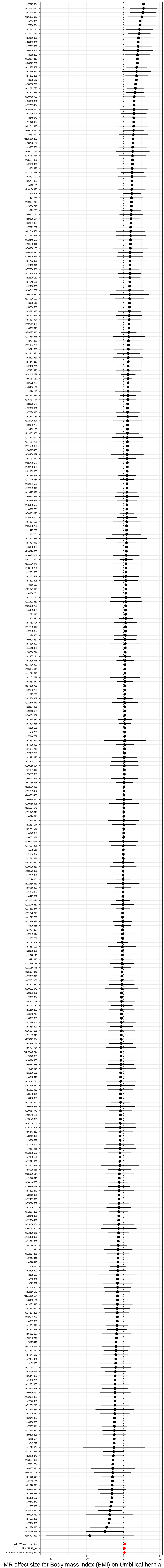
All - Weighted median
All - MR Egger
All - Inverse variance weighted
MR effect size for Body mass index (BMI) on Umbilical hernia

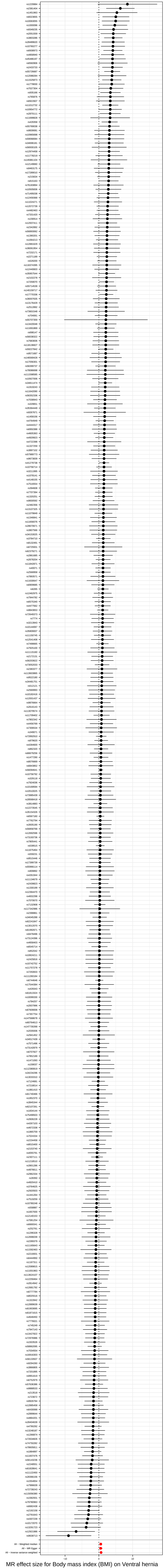
All - Weighted median
All - MR Egger
All - Inverse variance weighted
MR effect size for Body mass index (BMI) on Ventral hernia

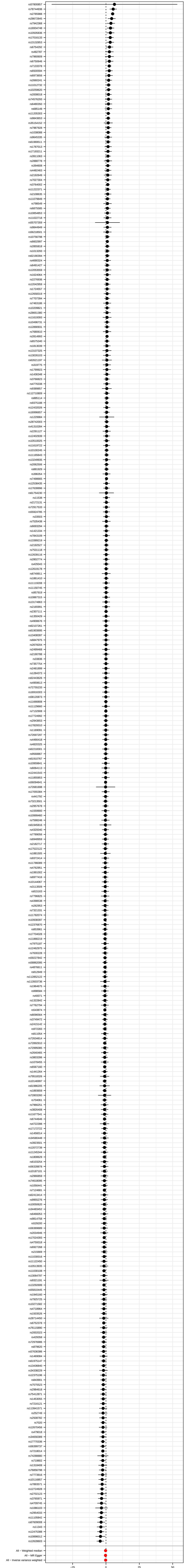

MR effect size for Body fat percentage on Inguinal hernia

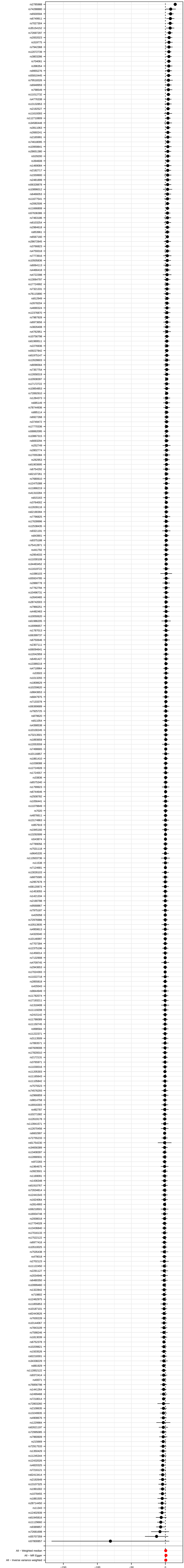

MR effect size for Body fat percentage on Umbilical hernia

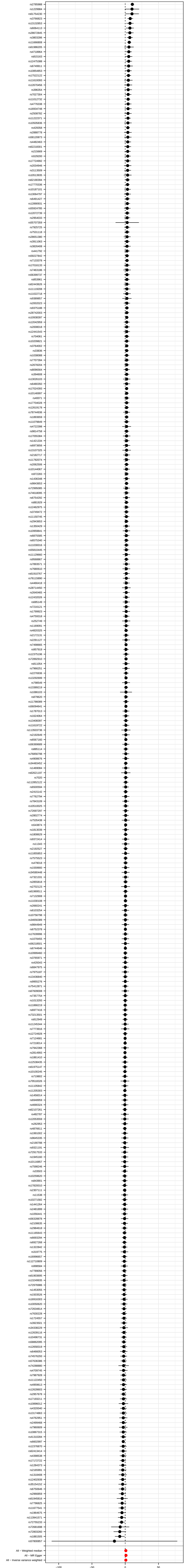

MR effect size for Body fat percentage on Ventral hernia

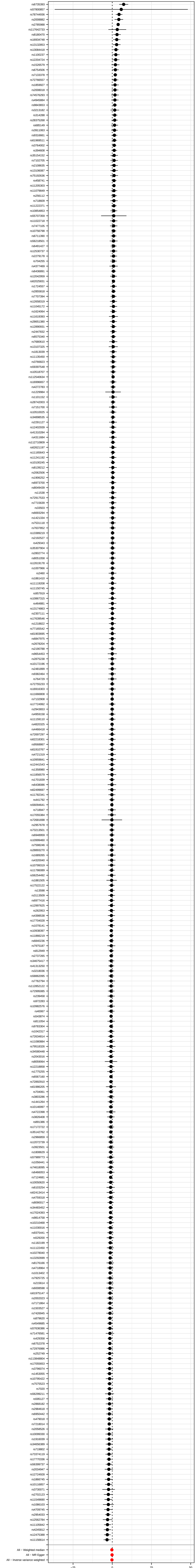

MR effect size for Trunk fat percentage on Inguinal hernia

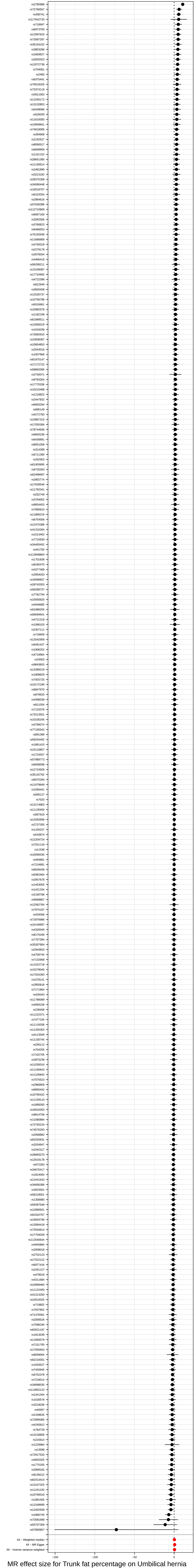

All − Weighted median
All − MR Egger
All − Inverse variance weighted
−150
−100
−50
0
MR effect size for Trunk fat percentage on Umbilical hernia

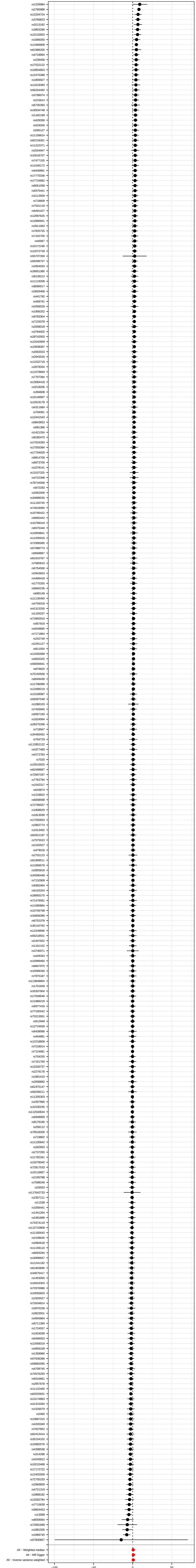

MR effect size for Trunk fat percentage on Ventral hernia

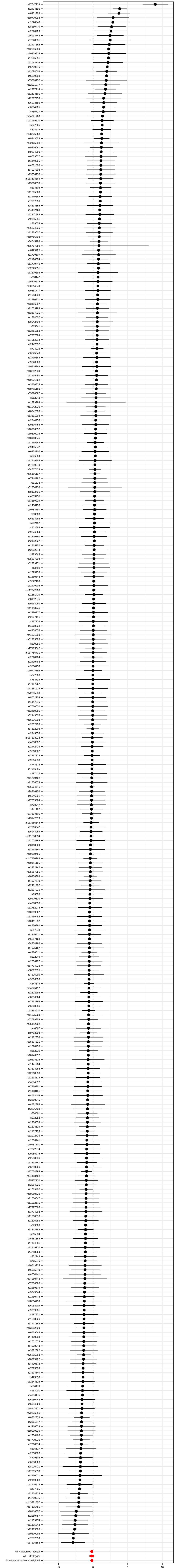

MR effect size for Trunk fat mass on Inguinal hernia

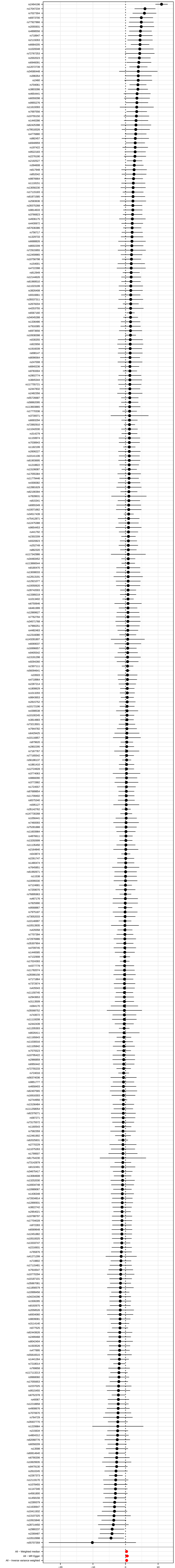

MR effect size for Trunk fat mass on Umbilical hernia

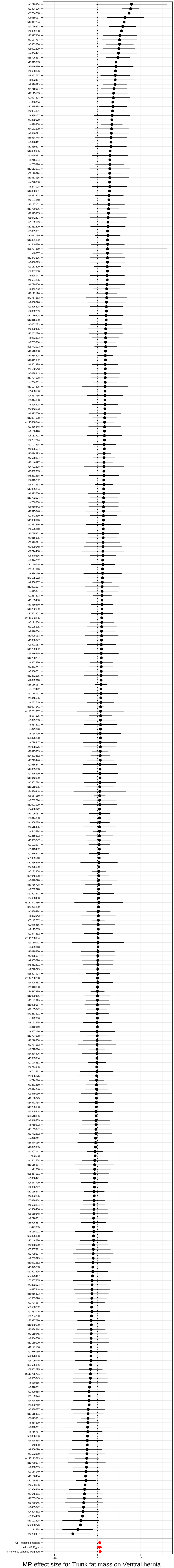
MR effect size for Trunk fat mass on Ventral hernia

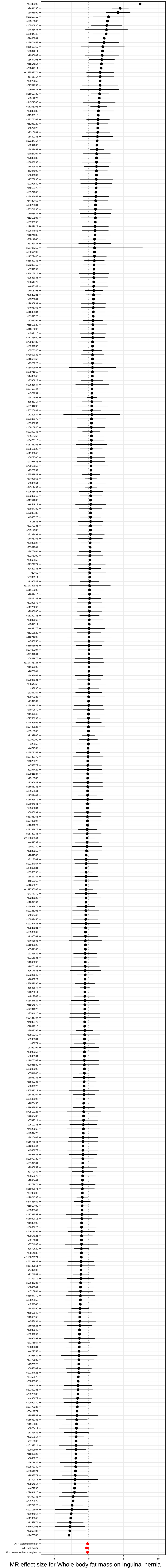

All − Weighted median
All − MR Egger
All − Inverse variance weighted
−5
0
5
10
MR effect size for Whole body fat mass on Inguinal hernia

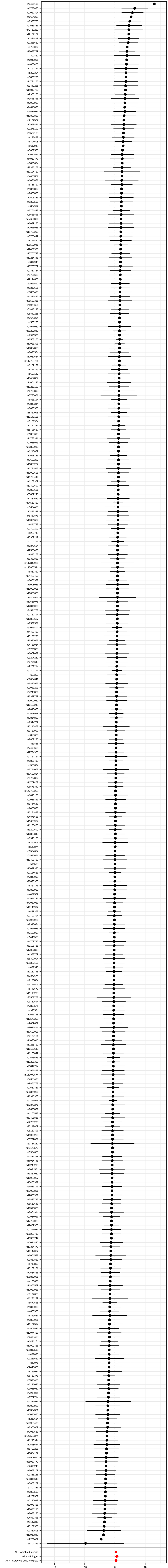

MR effect size for Whole body fat mass on Umbilical hernia

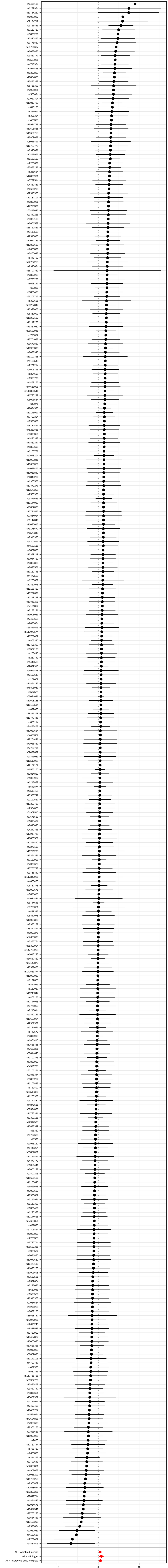

MR effect size for Whole body fat mass on Ventral hernia
