# Supplementary material for: Divergent associations between adiposity and hernia subtypes in European populations: A Mendelian randomization study
Source: Medicine (Baltimore). 2026 Oct 16;105(42):e51209. doi: 10.1097/MD.0000000000051209 (PMC13681110; doi:10.1097/MD.0000000000051209)
Supplement: Supplementary file 2 [file medi-105-e51209-s007.pdf]

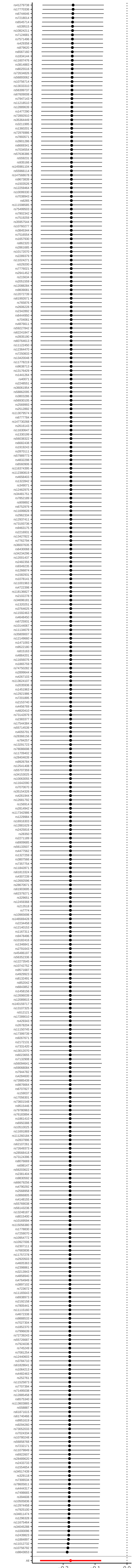

MR leave-one-out sensitivity analysis for
Body mass index (BMI) on Inguinal hernia

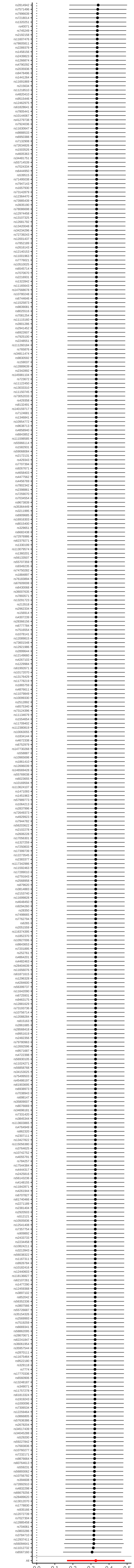

MR leave-one-out sensitivity analysis for
Body mass index (BMI) on Umbilical hernia

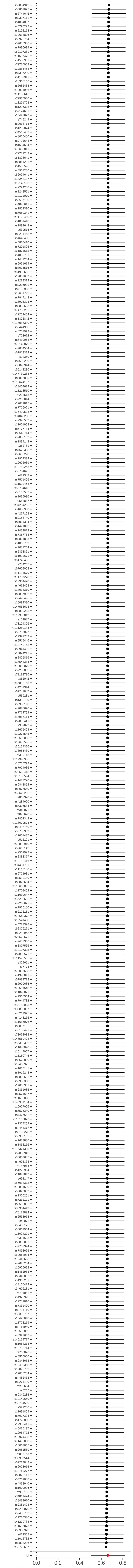

MR leave-one-out sensitivity analysis for
Body mass index (BMI) on Ventral hernia

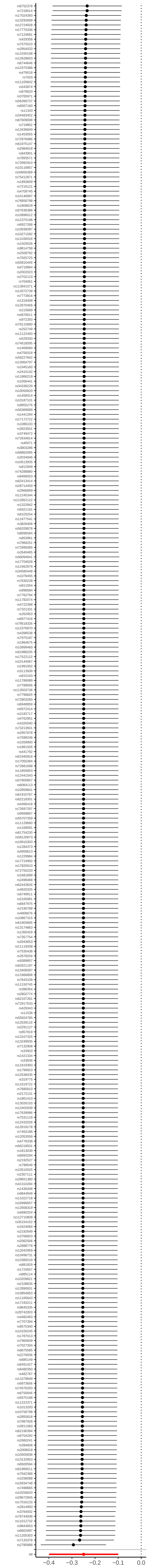

MR leave-one-out sensitivity analysis for
Body fat percentage on Inguinal hernia

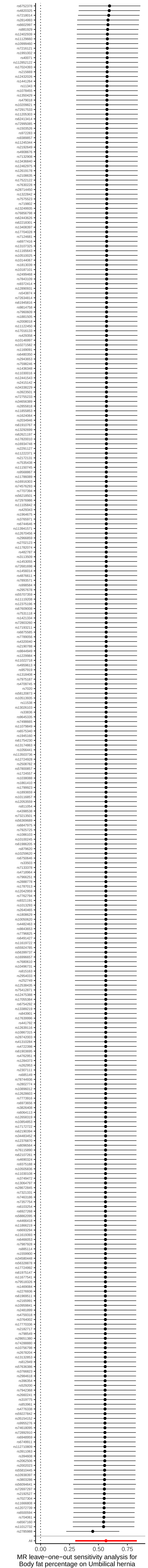

All
0.00
0.25
0.50
0.75
MR leave−one−out sensitivity analysis for
Body fat percentage on Umbilical hernia

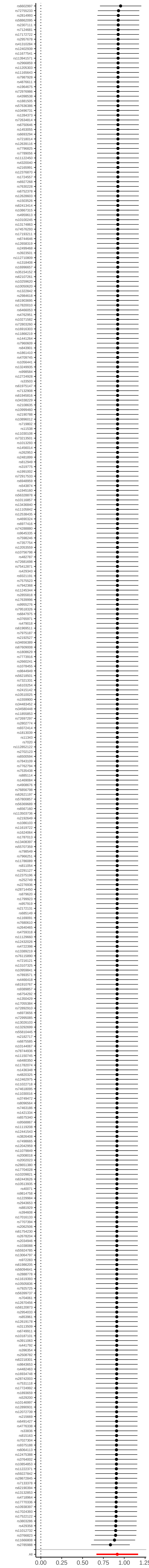

MR leave−one−out sensitivity analysis for
Body fat percentage on Ventral hernia

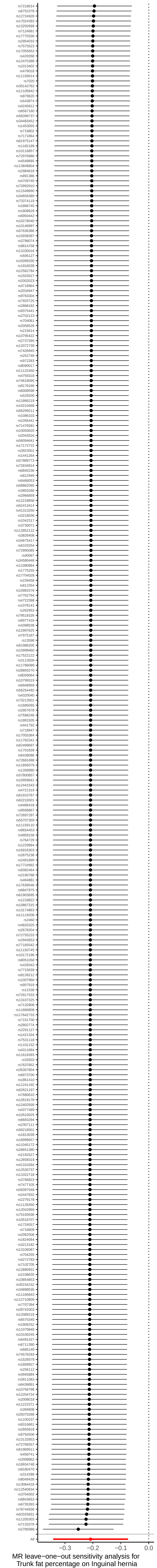

All
−0.3
−0.2
−0.1
0.0
MR leave−one−out sensitivity analysis for
Trunk fat percentage on Inguinal hernia

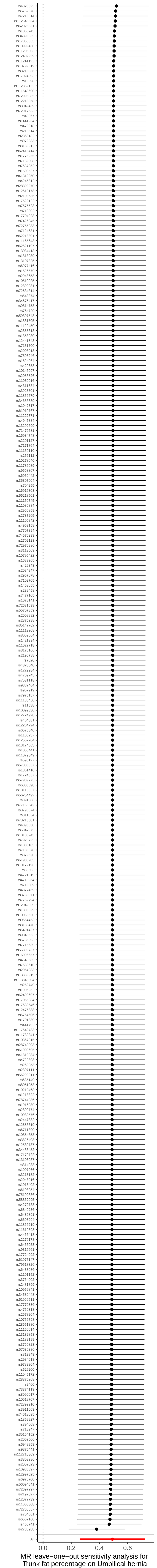
All
0.0
0.2
0.4
0.6
MR leave−one−out sensitivity analysis for
Trunk fat percentage on Umbilical hernia

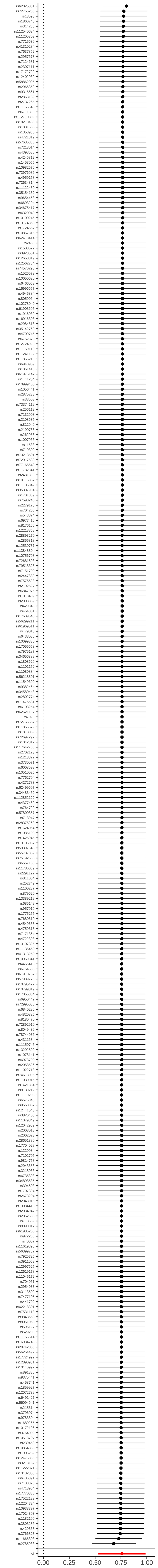

MR leave-one-out sensitivity analysis for
Trunk fat percentage on Ventral hernia

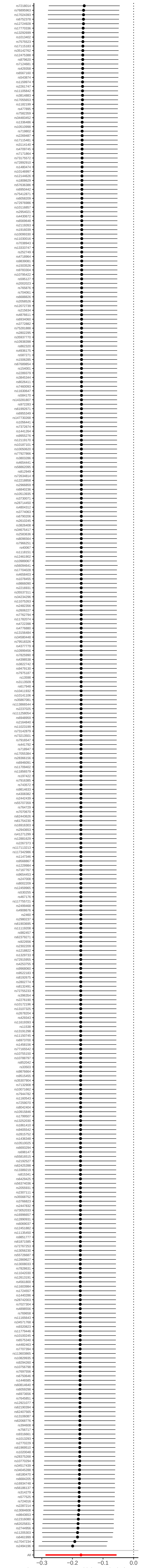

MR leave−one−out sensitivity analysis for
Trunk fat mass on Inguinal hernia

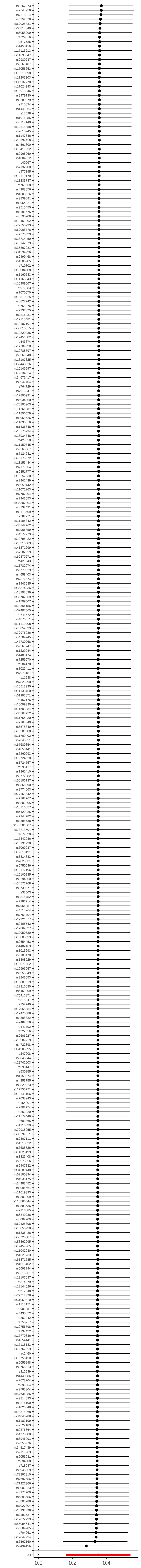

MR leave−one−out sensitivity analysis for
Trunk fat mass on Umbilical hernia

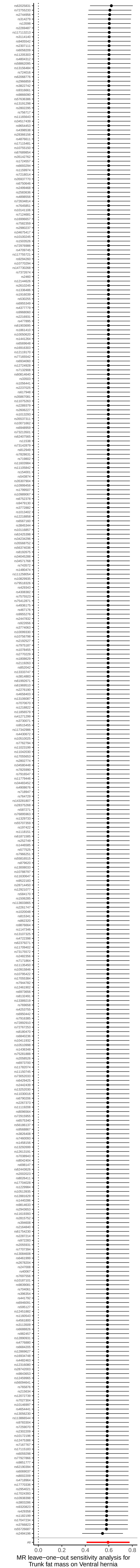
All
0.0
0.2
0.4
0.6
0.8
MR leave−one−out sensitivity analysis for
Trunk fat mass on Ventral hernia

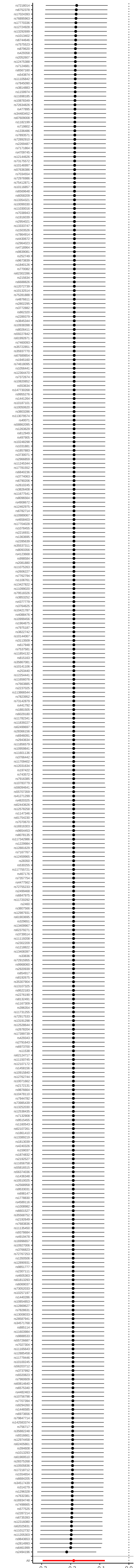

MR leave-one-out sensitivity analysis for
Whole body fat mass on Inguinal hernia

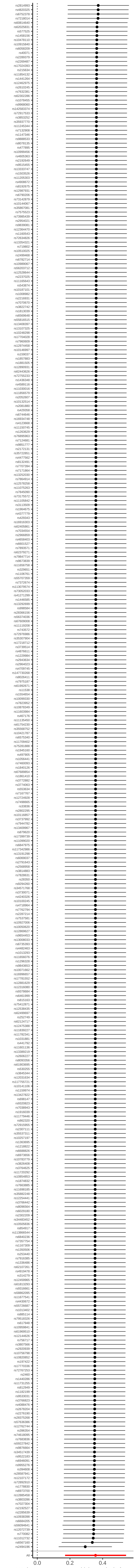

MR leave−one−out sensitivity analysis for
Whole body fat mass on Umbilical hernia

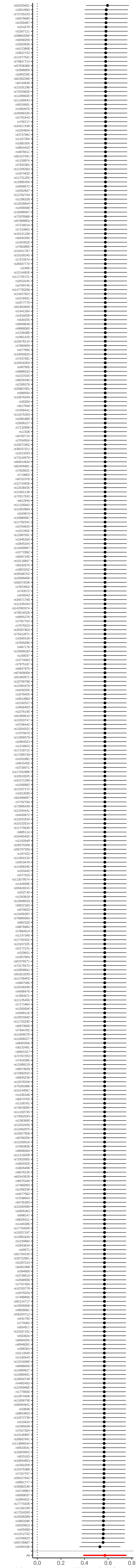

MR leave−one−out sensitivity analysis for
Whole body fat mass on Ventral hernia
